# Adaptive Servo-Ventilation: A Comprehensive Descriptive Study in the Geneva Lake Area

**DOI:** 10.3389/fmed.2020.00105

**Published:** 2020-04-03

**Authors:** Chloé Cantero, Dan Adler, Patrick Pasquina, Christophe Uldry, Bernard Egger, Maura Prella, Alain Bigin Younossian, Antoine Poncet, Paola Soccal-Gasche, Jean-Louis Pepin, Jean-Paul Janssens

**Affiliations:** ^1^Division of Pulmonary Diseases, Geneva University Hospitals (HUG), Geneva, Switzerland; ^2^Faculty of Medicine, University of Geneva, Geneva, Switzerland; ^3^Respiratory Diseases and Pulmonary Rehabilitation Center, Rolle Hospital, Rolle, Switzerland; ^4^Division of Pulmonary Diseases, Lausanne University Hospital (CHUV), Lausanne, Switzerland; ^5^Division of Pulmonary Diseases and Intensive Care, La Tour Hospital, Geneva, Switzerland; ^6^Center for Clinical Research and Division of Clinical Epidemiology, Department of Health and Community Medicine, University Hospitals of Geneva (HUG), Geneva, Switzerland; ^7^Inserm U1042 Unit, HP2 Laboratory, University Grenoble Alps, Grenoble, France; ^8^EFCR Laboratory, Thorax and Vessels and Vessels, Grenoble Alps University Hospital, Grenoble, France

**Keywords:** central sleep apnea, cheyne-stokes breathing, adaptive servo-ventilation, sleep-disordered breathing, emerging sleep apnea

## Abstract

**Background:** Use of adaptive servo-ventilation (ASV) has been questioned in patients with central sleep apnea (CSA) and chronic heart failure (CHF). This study aims to detail the present use of ASV in clinical practice.

**Methods:** Descriptive, cross-sectional, multicentric study of patients undergoing long term (≥3 months) ASV in the Cantons of Geneva or Vaud (1,288,378 inhabitants) followed by public or private hospitals, private practitioners and/or home care providers.

**Results:** Patients included (458) were mostly male (392; 85.6%), overweight [BMI (median, IQR): 29 kg/m^2^ (26; 33)], comorbid, with a median age of 71 years (59–77); 84% had been treated by CPAP before starting ASV. Indications for ASV were: emergent sleep apnea (ESA; 337; 73.6%), central sleep apnea (CSA; 108; 23.6%), obstructive sleep apnea (7; 1.5%), and overlap syndrome (6; 1.3%). Origin of CSA was cardiac (*n* = 30), neurological (*n* = 26), idiopathic (*n* = 28), or drug-related (*n* = 22). Among CSA cases, 60 (56%) patients had an echocardiography within the preceding 12 months; median left ventricular ejection fraction (LVEF) was 62.5% (54–65); 11 (18%) had a LVEF ≤45%. Average daily use of ASV was [mean (SD)] 368 (140) min; 13% used their device <3:30 h. Based on ventilator software, apnea-hypopnea index was normalized in 94% of subjects with data available (94% of 428).

**Conclusions:** Use of ASV has evolved from its original indication (CSA in CHF) to a heterogeneous predominantly male, aged, comorbid, and overweight population with mainly ESA or CSA. CSA in CHF represented only 6.5% of this population. Compliance and correction of respiratory events were satisfactory.

**Clinical Trial Registration:** www.ClinicalTrials.gov, identifier: NCT04054570.

## Introduction

Adaptive servo-ventilation (ASV) was proposed for managing Cheyne-Stokes breathing (CSB) in the late 90's, 20 years after the first report of treatment of obstructive sleep apnea syndrome (OSAS) by continuous positive airway pressure (CPAP) (1, 2). The initial algorithm was based on a fixed expiratory pressure (EPAP) and a variable pressure support, with the aim of controlling the “crescendo-decrescendo” of tidal volume and normalizing nocturnal breathing. Some devices targeted a minute ventilation set at 90% of spontaneous ventilation to allow nocturnal PaCO_2_ to increase slightly above the apnea threshold (3). Since their original design, options such as auto-titration of EPAP, of pressure support, and of back-up respiratory rate (BURR) have been added to the initial algorithms (4–8).

The recent SERVE-HF study questioned the use of ASV in cardiac failure: in patients with chronic heart failure (CHF) and a left ventricular ejection fraction (LVEF) ≤45%, ASV was associated with an increase in mortality (9). Although designed for CSB, use of ASV has drifted in clinical practice to other indications such as emerging sleep apnea (10–16), central or mixed apnea syndromes (17), either idiopathic, drug-induced (18–23), or associated with neurologic disorders (10–12, 24). Use of ASV in these indications does not rely on a high level of evidence of efficacy or benefit on outcomes such as survival or health-related quality of life. National (Facil-VAA, Clinicaltrials.gov registration No: NCT02835638) and multinational registries (READ-ASV, Clinicaltrials.gov registration No: NCT03032029) are ongoing to address this issue.

Very little information exists as to the present use of ASV in clinical practice in an unselected population (5, 25–27). The following study aims to describe the use of ASV in clinical practice in all patients within an area with a long-standing experience in home non-invasive ventilation (NIV) and treatment of sleep-disordered breathing (SDB) (28). The aims of this study are: 1/to detail “real life” present indications for ASV, and the prevalence of its use; 2/to describe the population under ASV, and its major comorbidities; 3/to provide detailed data on settings, compliance, correction of respiratory events and other items reported by ventilator software, as well as modalities of medical follow-up.

## Patients and Methods

### Study Design

A multicenter cross-sectional observational study performed in the Cantons of Geneva and Vaud (1,288,378 inhabitants in 2017) was designed to include all subjects under NIV followed by every possible structure involved: university hospitals, regional general hospitals, pulmonary rehabilitation centers, and pulmonologists in private practice.

Our definition of home NIV included all patients using bi-level positive pressure devices, multi-mode devices, ASV, volumetric ventilators, who were treated at home or in a long-term care facility (not a hospital) for ≥3 months. A cross-checking of patients treated in our area through health-care providers, and ventilator manufacturers guaranteed a comprehensive assessment of the targeted population.

Ethical approval was granted by the Cantonal Commission for Research Ethics (CCER) in Geneva, Switzerland (no. PB_2016-00925/15-275) in agreement with the amended Declaration of Helsinki. Trial was registered at clinicaltrials.gov (No: NCT04054570). Identification, screening, and data collection were performed by two investigators between June 1, 2016 and July 10, 2018.

### Inclusion/Exclusion Criteria

The present study focuses exclusively on patients treated by ASV. Patients were excluded if they used any other device, and if they (or their pulmonologist) refused to participate in this study.

### Data Collected

Anthropometric data, indication for ASV, major co-morbidities, pulmonary function tests, pulse-oximetry, arterial blood gases, ASV devices used, and information downloaded from devices were collected from medical records. No additional investigation was performed by the investigators. Availability of recent pulmonary function tests, pulse-oximetry, and arterial blood gases depended on “real-life” follow-up procedures and medical records. Results of echocardiographies were collected for patients with central sleep apnea (CSA) only. Data recorded were the most recent measurements performed within the 12 months prior to data collection.

### Statistical Analysis

Analyses included mainly descriptive statistics. Qualitative parameters were described as frequencies and percentages and quantitative parameters were described as mean and standard deviation (SD) or as median and quartiles (IQR).

Univariate and multivariate analyses regarding modality of follow up (hospital-based vs. private practitioners), and average daily use are described in on-line supplement (Table 1S).

Missing data were not replaced but simply reported, thus reflecting “real life” practices.

Statistical significance was assessed at the two-sided 0.05 level for all analyses. All analyses were performed using SPSS or R softwares (R Foundation for Statistical Computing, Vienna, Austria).

## Results

### Prevalence and Clinical Characteristics

Two university hospitals, one regional general hospital, one pulmonary rehabilitation center, and 38 of the 43 pulmonologists in private practice in the Cantons of Geneva and Vaud participated in data collection, i.e., all possible structures and specialists involved (albeit for 5 pulmonologists).

Of a total of 1,014 patients treated by home NIV, 500 (49%) had ASV devices (prevalence: 39/10^5^ inhabitants). By comparison, at the end of 2018, 19,350 patients were treated by CPAP in the same area (prevalence: 1,502/10^5^ inhabitants; ratio CPAP/ASV: 39:1) (personal communication, provided by health care providers listed in acknowledgments). Patients under ASV thus represent 2.5% of the population treated by CPAP for OSAS in the same area.

**Figure 1:**
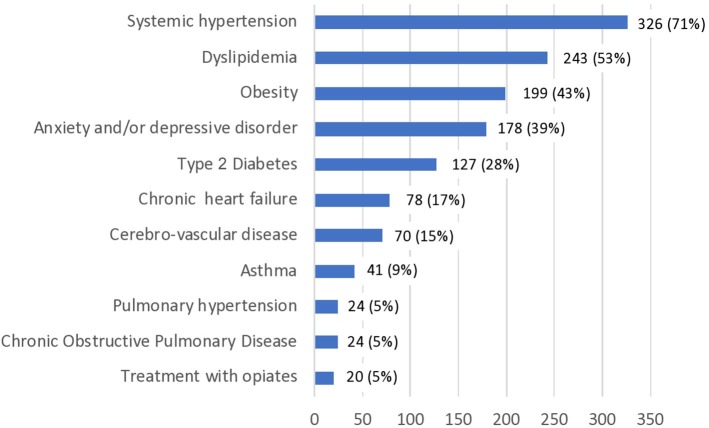
Comorbidities of population treated with adaptive servo-ventilation (*n* = 458).

Forty-two patients (8.4%) were excluded from the analysis of ASV devices (patient refusal: *n* = 21; refusal by treating pulmonologist: *n* = 21) (Figure 1S).

Clinical characteristics of the 458 patients under ASV and their co-morbidities are detailed in Table 1 and Figure 1. The population was rather old (median age 71 years; IQR [60; 76]), overweight (median body mass index (BMI) 29.1 kg/m^2^; IQR [26.3; 33.0]), with a strong male preponderance (*n* = 392; 85.6%), and a median of 3 comorbidities (IQR (2, 4)) (Figure 1). They had been treated by ASV for a median duration of 40 months (IQR [22; 62], range: 3 months−15 years).

**Table 1:** Characteristics of patients under adaptive servo-ventilation (ASV): *n* = 458 subjects.

	**All patients (*n* = 458)**	**Missing data**
**Anthropometric data**		
Age (years)	71 (60; 76)	–
Male	392 (85.6)	–
Body mass index (kg/m^2^)	29.1 (26.3; 33.0)	4
**Comorbidities**		
Systemic hypertension	326 (71)	–
Dyslipidemia	243 (53)	1
Obesity	199 (44)	2
Anxiety and/or depressive disorder	178 (39)	1
Type 2 diabetes	127 (28)	–
Chronic heart failure	78 (17)	1
Cerebrovascular disease	70 (15)	1
Asthma	41 (9)	1
Pulmonary hypertension	24 (5)	1
Chronic obstructive pulmonary disease	24 (5)	–
Opiate treatment	20 (4)	–

*Data are presented as N (%) or median and IQR (Q1; Q3)*.

### Devices

Devices used were: S9 AutoSet CS® (*n* = 268, 59%), AirCurve 10CS® (*n* = 137; 30%), AutoSet CS2® (*n* = 4; 1%), (by ResMed, San Diego, CA); BiPAP AutoSV Advanced System One® (*n* = 31; 7%), BiPAP AutoSV System One® (*n* = 6, 1%), BiPAP AutoSV Dreamstation® (*n* = 2) (by Philips Respironics, Murrysville, PA), and Prisma CR (*n* = 9, 2%) and SOMNOvent CR (*n* = 1) (by Löwenstein Medical Technology, Bad Ems, D), all in ASV or ASV-Auto modes.

### Indications

Indications for ASV are listed in Table 2. The majority of patients (*n* = 337, 73.6%) had emergent sleep apnea; 108 (23.6%) had central sleep apnea (CSA); 13 had atypical indications for ASV (OSAS or overlap syndrome), 6 of whom were considered as CPAP “failures.” Most patients (*n* = 385,84%) had been treated by CPAP before starting ASV.

**Table 2:** Indications for adaptive servo-ventilation (*n* = 458).

	**All patients (*n* = 458)**	**Prior use of CPAP**	**Echocardiography^b^**
Indications			–
Emergent sleep apnea	337 (73.6)	337/337 (100)	–
Central sleep apnea syndrome	108 (23.6)	42/108 (39)	60/108 (56)
Obstructive sleep apnea	7 (1.5)	6/7 (86)	–
Overlap syndrome (COPD and OSA)	6 (1.3)	0	–
Total	458	385/458 (84%)	–
Causes of central sleep apnea syndrome^a^	(*n* = 108)	(*n* = 42)	(*n* = 60)
Cardiac origin	20	4	17
Cardiac and neurologic origin	9	3	8
Cardiac and medication	1	0	1
Neurological disorder	16	8	7
Neurological disorder and medication	1	1	0
Medication (opiates)	22	9	5
Idiopathic	28	14	21
No information available	11	3	1

*CPAP, Continuous positive airway pressure; ASV, Adaptive servo-ventilation*.

a *Fifteen patients had typical Cheyne-Stokes breathing (7 “cardiac,” 7 “idiopathic” and 1 “neurologic” origin)*.

b *Follow-up echocardiography was recorded only in patients with central sleep apnea syndrome*.

*LVEF estimated by echocardiography was ≤45% in 11 patients (in all cases, presumed cause of central sleep apnea was of “cardiac” or “cardiac and neurologic” origin). Median LVEF was 62.5% (IQR 54–65)*.

Among CSA patients, only 30 (28%) had CSA of cardiac origin and 15 (14%) had typical CSB.

Patients reported as “*CSA related to neurological disorders”* (*n* = 26) had the following diagnoses: cerebro-vascular disease (*n* = 21), various CNS tumors (meningioma, astrocytoma, pituitary adenoma; *n* = 3), multiple sclerosis (*n* = 1), myasthenia gravis (*n* = 1), hereditary spinocerebellar ataxia (*n* = 1). Several diagnoses were sometimes present simultaneously. Causal relation between CSA and associated neurological disorders was presumptive.

Patients reported as “*CSA related to medication”* were under opiates (methadone, morphine sulfate), administered either for chronic pain (*n* = 2), or for substitutive therapy of prior opiate addiction (*n* = 20), often combined with benzodiazepines (bromazepam, alprazolam, oxazepam, lorazepam) or zolpidem.

Despite usual screening for cardiac and/or neurological disorders in this population, 28 cases of CSA (25.9%) were considered idiopathic.

### Settings of Ventilators and Interfaces

Interfaces most commonly used were facial masks (*n* = 318, 69%), followed by nasal masks (*n* = 93, 20%) and nasal pillows (*n* = 45, 10%) (Table 3A). Combining all devices, 158 (35%) used a variable EPAP, and 289 (65%) a fixed EPAP (*n* = 11: missing data). Minimal pressure support was most often the “default value” provided by the manufacturer (i.e., 3 cmH_2_O for ResMed® devices, and 0 cmH_2_O for Philips Respironics® and Lowenstein Medical® devices) (Table 3B).

**Table 3:** ASV ventilator settings (*n* = 458), interfaces, humidifiers, and oxygen supplementation.

**A. Interfaces, humidifier, and oxygen supplementation (n, %)**		**Missing data**
*Interfaces*		2
- Facial masks - Nasal masks - Nasal pillows	318 (70) - 93 (20) - 45 (10)	
Humidifier	314 (69)	1
Oxygen supplementation during ASV	16 (3.5)	–
**B. Ventilator settings (ASV)**		
	**ResMed**^®^ devices^**^	**Philips respironics**^®^ **and lowenstein**^®^ **devices^§^**
All values are median (IQR)	*n* = 409	*n* = 49^*^
Minimal pressure support (cmH_2_O)	3 (3; 3)	0 (0; 3)
Maximal pressure support (cmH_2_O)	10 (10; 12)	10 (8; 15)
*Fixed EPAP*	*n = 284*	*n = 5*
Fixed EPAP (cmH_2_O)	6 (5; 8)	9 (8; 10)
*Variable EPAP*	*n = 115*	*n = 43*
Minimal EPAP (cmH_2_O)	5 (4; 7.5)	6 (4; 9.7)
Maximal EPAP (cmH_2_O)	11 (10; 14)	12 (10; 14.5)
Back-up respiratory rate (Cycles/min)	–	12 (12; 13.7)
Missing data	10	1

*EPAP: Expiratory positive airway pressure*.

** *See text for details. Default EPAP value set at 3 cmH_2_O*.

§ *See text for details. Default EPAP value set at 0 cmH_2_O*.

* *26 patients in “Auto” mode for back-up respiratory rate*.

### Monitoring

Echocardiography (Table 2): of all patients with CSA, 60 (56%) patients had an echocardiographic estimation of LVEF within the preceding 12 months; this included 26 of the 30 patients with cardiac disease (87%). Median value (IQR) for LVEF was in the normal range (62.5% [54; 65]), although it was ≤45% in 11 cases.

Data downloaded from the ASV devices (software: Rescan®, ResMed; Encore Basic®, Philips Respironics, PrismaTS®, Weinmann support®, and PrismaTS®, Lowenstein Medical®) covered a median period of 90 days (IQR: [30; 182]) (Tables 3B, 4).

**Table 4:** Data retrieved from ventilators and monitoring of ASV treatment (*n* = 458).

	**ResMed^®^ devices**	**Philips respironics^®^ and lowenstein medical^®^ devices**
All values are median (IQR) unless stated otherwise	*n* = 409	*n* = 49
EPAP (cmH_2_O)	6.5 (5.2; 8.5)	–
IPAP (cmH_2_O)	10.7 (9.2; 12.7)	–
EPAP 90% (cmH_2_O)	–	9.2 (7.0; 10.7)
Pressure support (cmH_2_O)	–	2.8 (1.8; 4.7)
Tidal volume (ml)	440 (380; 520)	486 (416; 567)
Minute ventilation (L/min)	6.6 (5.8; 7.9)	8.1 (6.7; 9.2)
Leaks, unintentional (L/min)	0.0 (0.0; 3.6)	–
Leaks, 95th centile, unintentional (L/min)	9.6 (2.4; 21.6)	–
Leaks (total) (L/min)	–	37 (30; 42)
Apnea index (N/h)	0.1 (0.0; 0.6)	0.7 (0.3; 2.0)
Apnea-hypopnea index (N/h)	1.2 (0.4; 3.1)	3.5 (1.8; 5.9)
Mean daily use (SD; min/day)	366 (143)	383 (120)

*All values are reported as Median (IQR) unless specified otherwise. Data from ResMed® devices, Philips Respironics®, and Lowenstein Medical® devices are reported differently by their specific software and are thus shown separately. Unintentional leaks: leaks not related to expiratory valve of mask or circuit. Total leaks: sum of intentional (related to mask expiratory valve) and unintentional leaks. EPAP, Expiratory positive airway pressure; IPAP, Inspiratory positive airway pressure*.

### Compliance

**Figure 2:**
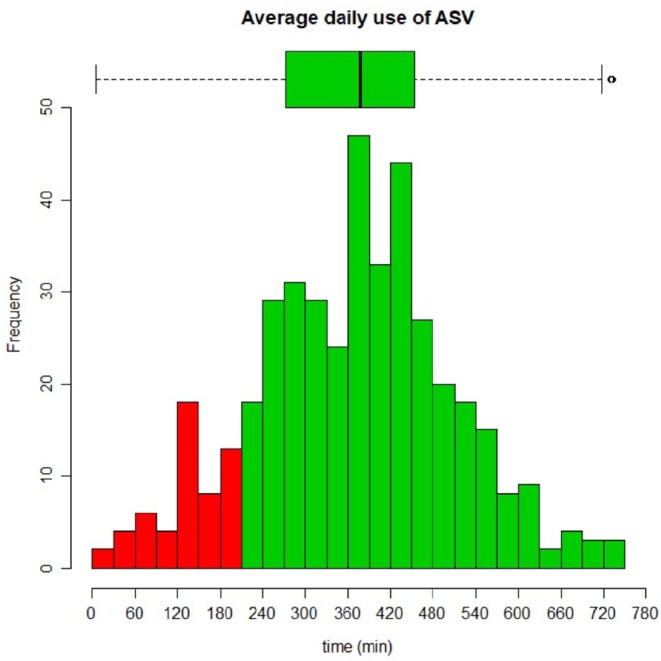
Distribution of compliance (average daily use of ASV) among 458 subjects. Average use was 368 ± 140 min.

Average use of ASV [available in 419 (91%) patients] was above 6 h/night [mean (SD): 368 (140) min]; 13% (55/419) of all patients used their device <3:30 h (Figure 2). See on-line supplement for items associated with average daily use, and Table 2S for daily use by diagnostic category.

**Figure 3:**
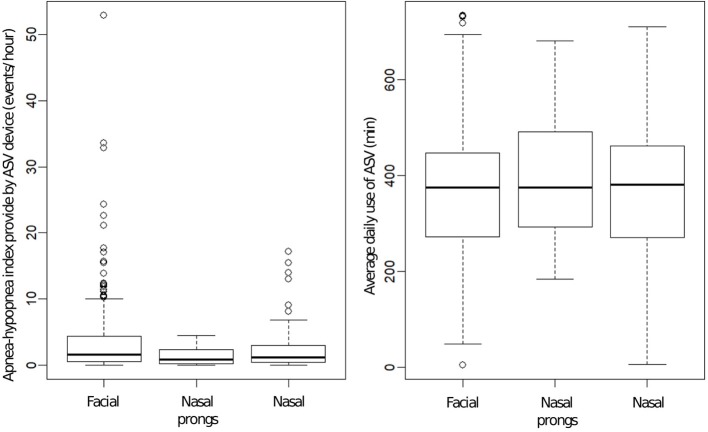
**(Left)** Box plots of apnea-hypopnea index (AHI) provided by ventilator software according to interface used (data were available for 428 patients, missing for 30). Differences were statistically significant (*p* = 0.012) but not clinically relevant. Bold line is median value; box defines 25th and 75th centiles. **(Right)** Box plots of average daily use of adaptive servo-ventilation according to interface used (data available for 419 patients, missing for 39). Values for daily use and AHI were average values over 90 days (median = 90; IQR: [32; 182]).

Choice of interface was not associated with any significant difference in average daily use (facial mask: 365 ± 142 min; nasal masks: 368 ± 138 min; nasal prongs: 392 ± 128 min; *p* = 0.53) (Figure 3).

### AHI and Other Data Provided by Ventilator Software

Relevant data retrieved from ventilators are described by device in Table 4. Apnea-hypopnea index (AHI) under ASV (available in 428 patients) was normalized (i.e., <10/h) in 94% (*n* = 401). AHI was slightly lower with nasal masks, than with facial or nasal pillows: this difference had no clinical relevance (Figure 3). Table 2S provides AHI by diagnostic category.

Leaks (available in 395 patients) were under threshold values suggested as relevant by manufacturers (24 L/min for unintentional leaks, ResMed devices; 55 L/min for total leaks, Philips Respironics devices) in 364 (92%) patients (Table 4).

### Nocturnal Pulse Oximetry

Of the 458 patients included, 209 had had a nocturnal pulse oximetry performed under ASV within the 12 months prior to data collection. Mean SpO_2_ value was: 93.3 (2.4) (available for *n* = 202); median SpO_2_ value was 94% (93; 96) (*n* = 198); median time spent with an SpO2 <90% was 0.4% of total recording time (IQR: 0.0; 5.7); median oxygen desaturation index (>3%) was 6.7/h (3.0; 14.9).

### Follow-Up (Hospital-Based vs. Private Practitioners)

Data are provided in on-line supplement.

## Discussion

The present report is to our knowledge the largest report of unselected patients treated by long term ASV. Prevalence of ASV is presently very close to that of NIV for chronic hypercapnic respiratory failure in our area. The ASV population represents 2.5% of the population treated by CPAP for sleep-disordered breathing (SDB). The typical profile of the “ASV patient” in this study is an overweight or obese male subject in his early 70's, with emerging sleep apnea uncontrolled by CPAP, and several cardiac, respiratory and/or neurological comorbidities. Compliance is higher than previously reported, with a very good control of SDB, based on data downloaded from the ASV devices. Our data show that ASV devices are prescribed in clinical situations which extend far beyond the initial target of ASV, i.e., Cheyne-Stokes Breathing (CSB): indeed, 4 years after the publication of the SERVE-HF study (9), CSB in CHF represents only 1.5% of the population treated by ASV, and only 6.6% of all subjects on ASV (30/458) have CSA related to CHF. In spite of SERVE-HF (9), a third (36%) of cardiac patients under ASV still had a LVEF ≤45%. While all but 4 patients with CHF (26/30; 87%) had undergone an echocardiography within the prior 12 months, this was the case for only 56% (*n* = 60/108) of the whole ASV population treated for CSA. ASV patients are mostly followed by pulmonologists in private practice (67%). Outcomes (AHI, compliance) are similar irrespective of follow-up by a pulmonologist in private practice or hospital-based (On-Line Supplement).

The majority of patients were prescribed ASV for emergent or persistent CSA (74%) under CPAP. Using a large population-based sample of CPAP device data, the dynamic nature of CSA occurring during CPAP therapy has been recently highlighted. A big data analysis (*n* = 133,006) using telemonitoring data of US CPAP devices identified a variety of CSA trajectories occurring during CPAP therapy (29). Previous studies in the field with relatively small sample sizes and heterogeneous populations in terms of titration procedures provided inconsistent results (30). The Liu et al. “real life” analysis allowed to delineate the true prevalence of CSA at 3.5% of CPAP treated OSA. CSA was transient, persistent, or emergent in 55.1, 25.2, and 19.7%, respectively (29). CSA under CPAP, whatever the subtype, was also clearly associated with a higher risk of therapy discontinuation (29) suggesting that these patients should be identified and specific phenotypes associated with emergent CSA more clearly described. These figures are in accordance with our results: close to 20,000 patients are currently treated by CPAP in our area: subjects under ASV represent 2.5% of those under CPAP, and most of them were previously under CPAP. In another big data analysis, patients demonstrated a better control of residual events and an improvement in adherence early after switching from CPAP to ASV (14). Compliance and AHI levels in this study are in agreement with these observations.

The ASV population described differs from previous reports, which either focus on CHF (9, 31), or on specific neurological disorders (Chiari malformation (32), Multiple System Atrophy (33), opiate or other drug toxicities (16, 21, 22, 34, 35). Two retrospective studies (25, 26) describe relatively large populations under ASV. Carnevale et al. (25) included 74 patients: 33 with CHF and 41 with various neurological disorders or idiopathic CSA. ASV was effective in reducing AHI, sleepiness, and improving SpO_2_ in both groups. A German study analyzed 285 patients receiving ASV and undergoing diagnostic polysomnography (PSG). The most common indications were CHF and emergent CSA (67%), CSA in CHF (22%), idiopathic CSA (10%), and drug-induced CSA (0.4%) (26). The patients described in our study are long term users of ASV [median 40 months, IQR (22; 62)] which suggests at least a subjective benefit (although no formal assessment of health-related quality of life or symptom score were performed during this study). Interestingly, compliance rates are high (Figures 2, 3, Table 4, Table 2S), and treatment of underlying SDB is effective based on data downloaded by the ventilators (IAH) and nocturnal pulse oximetry. Correlation between AHI from ventilator software and PSG, in the absence of major leaks, has been reported as appropriate for clinical use (36–38).

An interesting group of patients is that of drug-induced SDB. These patients are under opioids, either for treatment of chronic pain or for substitutive treatment of prior drug abuse, often in combination with benzodiazepines. This population is rapidly increasing, reaching epidemic proportions in the USA (39). OSA, CSA, and nocturnal hypoventilation have all been reported under long term opiate usage (21, 22, 35). ASV controls opiate-induced CSA in 58% of patients: it is more effective than CPAP, and equivalent to bi-level positive pressure ventilation (22). Patients under ASV for opiate-induced SDB in the present study were treated for central SDB. Prevalence of SDB is known to be high in these patients and is often symptomatic (40, 41). Although a few clinical studies have shown that ASV could control SDB (CSA) in patients under chronic high dose opioid treatment (18, 19, 42), there are no randomized trials showing a long term improvement in either symptom scales, HRQL or survival. Many questions therefore remain, such as: should we screen patients under opioids for SDB? Should treatment be proposed systematically? What threshold levels of AHI are relevant? Before the use of ASV explodes in this population, with the associated costs, these issues warrant prospective trials.

For patients with CHF, SERVE-HF has radically changed the clinical approach to CSB in CHF. In this study, a significant percentage of patients with CHF, LVEF ≤45% and SDB remain under ASV (11/30: 37% of all cardiac cases). Almost all of these patients had an echocardiography within the preceding 12 months. This shows that despite SERVE-HF, a certain number of unwarranted prescriptions of ASV persist. The data collected do not allow us to determine to what extent ASV treatments were interrupted after SERVE-HF, or how many patients who are within the high-risk population identified by SERVE-HF, requested to remain under ASV because of improvement of their symptoms (43).

This study has several limitations: first of all, it is a descriptive study and must not be considered as a validation of ASV in the indications reported. Our aim was to better define practices regarding ASV and to identify subgroups of interest for whom prospective randomized studies should be conducted (i.e., CSA related to medication, opiates, CSA associated to neurological disorders, or “idiopathic” CSA). Secondly, there are missing values in our data collection: all result of the “real life” design of this study and reflect the variability of follow-up of ASV patients. Thirdly, other than compliance and AHI, there are no outcome measures and no subjective scores. It was the aim of this study to describe a new population in the field of home ventilatory support, and indeed, the results provide a new insight to the use of ASV in chronic home care and future areas of research. Finally, we recorded echocardiographic data only in patients corresponding to the SERVE-HF trial inclusion criteria (i.e., subjects with “*de novo*” CSA excluding patients under prior CPAP and thus ESA) (9) to determine to what extent patients with a LVEF <45% in this specific group of ASV users were still being treated. Because left ventricular dysfunction and chronic heart failure have also been documented in patients with emergent sleep apnea (ESA), the prevalence of cardiac dysfunction in the ASV population studied may be underestimated (26, 31, 44). Indeed 13% of ESA patients had a history of chronic heart failure and cardiovascular risk factors were highly prevalent. In a recent French multicentric descriptive study of 177 patients under ASV, 20.3% had emergent CSA: 75% of these patients had a cardiac disease and 14% had a LVEF <45% (45). Future studies should focus on ESA, clinical and echocardiographic findings in this group and prognosis under ASV.

In summary, use of ASV targets a much wider population than that of CSB in CHF. Although there are rationales for proposing ASV in the different populations described, and data presented suggest that ASV is effective in these groups (excellent compliance, correction of SDB according to AHI), SERVE-HF has shown to what extent evidence can be counter-intuitive. Because of the numeric importance of some of the patient groups (i.e., chronic opioid treatments or addiction in the US), future prospective randomized controlled trials must explore benefits of ASV in terms of symptom control, HRQL, and survival. The description of the population treated by ASV is a first step to initiate prospective randomized studies and validate—or not—the use of ASV in specific indications.

## Data Availability Statement

The datasets generated for this study are available on request to the corresponding author.

## Ethics Statement

The studies involving human participants were reviewed and approved by Ethical approval was granted by the Cantonal Commission for Research Ethics (CCER) in Geneva, Switzerland (no. PB_2016-00925/15-275) in agreement with the amended Declaration of Helsinki. The patients/participants provided their written informed consent to participate in this study.

## Author Contributions

CC, PP, and J-PJ contributed conception and design of the study. CC and PP organized the database. AP performed the statistical analysis. CC and J-PJ wrote the first draft of the manuscript. CC, PP, DA, and J-PJ wrote sections of the manuscript. All authors contributed to manuscript revision, read, and approved the submitted version.

### Conflict of Interest

The authors declare that the research was conducted in the absence of any commercial or financial relationships that could be construed as a potential conflict of interest.
