# Supplementary material for: Adaptive Servo-Ventilation: A Comprehensive Descriptive Study in the Geneva Lake Area
Source: Front Med (Lausanne). 2020 Apr 3;7:105. doi: 10.3389/fmed.2020.00105 (PMC7145945; doi:10.3389/fmed.2020.00105)
Supplement: Supplementary file 1 [file Table_1.docx]

Table 1S. Average daily use of ASV analysis

|  | Descriptive statistics (n=419) | | | Univariate analysis | | Multivariate analysis* | |
| --- | --- | --- | --- | --- | --- | --- | --- |
|  | Modality | Total | Mean (SD) | Mean diff (95%CI) | p value | Beta (95%CI) | p value |
| Interface^1^ | Facial | 292 | 365 (142) | - |  | - |  |
|  | Nasal | 87 | 368 (138) | 3 (-31, 36) | 0.876 | 8 (-24, 41) | 0.619 |
|  | Nasal pillows | 40 | 392 (128) | 27 (-20, 73) | 0.260 | 50 (4, 95) | 0.031 |
| Prior treatment with CPAP | No | 62 | 369 (145) | - |  | - |  |
|  | Yes | 353 | 367 (139) | -3 (-40, 35) | 0.894 | -2 (-41, 37) | 0.917 |
| Gender | Female | 62 | 344 (143) | - |  | - |  |
|  | Male | 357 | 373 (139) | 28 (-9, 66) | 0.142 | 38 (-2, 77) | 0.060 |
| Metabolic syndrome | No | 88 | 335 (127) | - |  | - |  |
|  | Yes | 331 | 377 (142) | 42 (9, 75) | 0.012 | -5 (-42, 31) | 0.770 |
| Obesity | No | 235 | 353 (133) | - |  | - |  |
|  | Yes | 182 | 389 (147) | 36 (9, 63) | 0.009 | 25 (-2, 52) | 0.071 |
| Chronic heart failure | No | 348 | 356 (138) | - |  | - |  |
|  | Yes | 70 | 429 (133) | 73 (37, 108) | <0.001 | 58 (19, 96) | 0.003 |
| Cerebro-vascular disease | No | 356 | 369 (140) | - |  | - |  |
|  | Yes | 62 | 366 (137) | -3 (-41, 35) | 0.866 | -23 (-62, 16) | 0.252 |
| Anxio-depressive disorders | No | 260 | 365 (132) | - |  | - |  |
|  | Yes | 158 | 374 (152) | 8 (-19, 36) | 0.554 | 22 (-6, 51) | 0.122 |
| Use of opioids | No | 402 | 372 (139) | - |  | - |  |
|  | Yes | 17 | 277 (134) | -95 (-163, -28) | 0.006 | -59 (-131, 12) | 0.105 |
| Time under ASV (months) | [0,12) | 56 | 336 (137) | - |  | - |  |
|  | [12,36) | 117 | 353 (141) | 17 (-27, 61) | 0.450 | 4 (-39, 47) | 0.860 |
|  | [36,72) | 171 | 381 (137) | 45 (3, 87) | 0.035 | 35 (-6, 77) | 0.093 |
|  | [72,341] | 75 | 389 (140) | 53 (5, 101) | 0.031 | 23 (-26, 71) | 0.358 |
| Age | Per decade |  |  | 27 (16, 37) | <0.001 | 23 (10, 36) | <0.001 |

Data available for 419 patients (n=39 missing data)

*Multivariate analysis was performed on a total of 402 patients due to 17 additional missing data (total missing data: n=56)

1: Overall effect: type of mask (p=0.53); time under ASV (p=0.056)
