# Supplementary material for: Adaptive Servo-Ventilation: A Comprehensive Descriptive Study in the Geneva Lake Area
Source: Front Med (Lausanne). 2020 Apr 3;7:105. doi: 10.3389/fmed.2020.00105 (PMC7145945; doi:10.3389/fmed.2020.00105)
Supplement: Supplementary file 2 [file Data_Sheet_1.docx]

**On-line supplement**

**Adaptive servo-ventilation: a comprehensive descriptive study in the Geneva Lake area.**

C. Cantero, MD^1^; D. Adler, MD^2,3^; P. Pasquina^2^; C. Uldry, MD^4^; B. Egger, MD^4^; M. Prella, MD^5^; A.B. Younossian, MD^6^; A. Poncet, MSc^3,7^; P. Gasche, MD^2,3^; JL Pépin, MD^8,9^; JP Janssens, MD^2,3^

On behalf of all the investigators of the Geneva Lake Study

Table of contents:

Flow chart

*Statistical methods*

Items associated with average daily use: methods

Items associated with follow-up (hospital-based vs. private practicioner)

*Results*

Items associated with average daily use: results

Items associated with follow-up (hospital-based vs. private practicioner): results

*Statistical methods:*

Items associated with average daily use: statistical methods

A search of covariates independently associated with average daily use of ASV was performed using a multivariate linear regression model. A list of parameters comprising patient characteristics, comorbidities, type of interface and time under ASV was defined *a priori*, based on clinical relevance, and all of them were included in the model. Model assumptions were assessed using diagnostic plots (1. Residuals vs. fitted, 2. Normal Q-Q plot, 3. Spread location plot and 4. Residual vs. Leverage).

Items associated with follow-up (hospital-based vs. private practitioner)

To determine whether modality of follow up (hospital-based vs private practitioners) was associated with specific indicators (medical evaluation with the preceding 12 months, number of comorbidities, compliance, residual AHI), we performed chi-square tests or Fisher exact tests for qualitative parameters and Student tests or Mann-Whitney tests for quantitative parameters, as appropriate.

*Results*

Items associated with average daily use

In multivariate analysis, average daily use was independently associated with CHF and age (see Table 1S). Use of ASV was one hour higher in patients with CHF (beta (95%CI) = 58 (19, 96) min, p=0.003) and increased by 23 minutes for every increment of 10 years in age (beta = 23 (10, 36) min, p<0.001).

Items associated with follow-up (hospital-based vs. private practitioner)

Medical follow-up for ASV was provided exclusively by pulmonologists in private practice for 309 patients (67%), and by one of the 4 participating hospitals in 149 patients (33%) (Of whom 36 subjects were also followed by a private pulmonologist). When comparing patients according to modalities of follow-up (hospital-based vs private practitioners), the following characteristics exhibit no differences:

- number of comorbidities, mean (SD): 2.8 (1.7) vs. 3.0 (1.6), p=0.268;
- percentage of patients evaluated within the last 12 months: 86% in both groups, p=1;
- average daily use (min), mean (SD): 361 (139) vs 372 (140), p=0.460;
- residual AHI (/hour), mean (SD): 3.6 (6.2) vs 2.9 (4.1), p=0.234

However, Hospitals used variable EPAP modes (ASV-auto) more frequently (50% vs 27% for private practitioners, p<0.001).
