# Supplementary figures and images for: Adaptive Servo-Ventilation: A Comprehensive Descriptive Study in the Geneva Lake Area
Source: Front Med (Lausanne). 2020 Apr 3;7:105. doi: 10.3389/fmed.2020.00105 (PMC7145945; doi:10.3389/fmed.2020.00105)

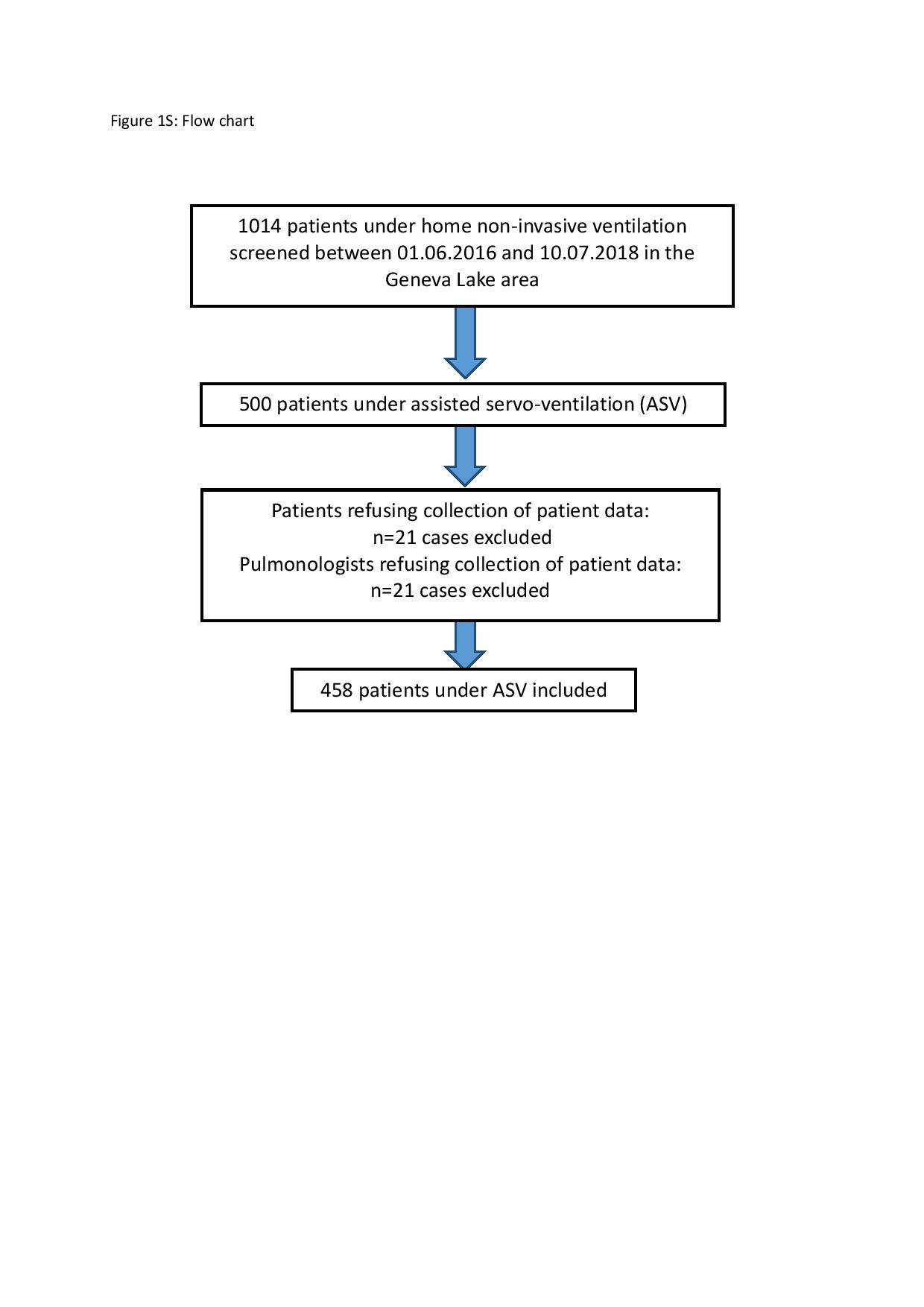

Supplement: Supplementary file 3 [file Image_1.jpeg]
